# Earthquake forecasting model for Nepal to improve prevention

**DOI:** 10.3126/nje.v7i4.20626

**Published:** 2017-12-31

**Authors:** Brijesh Sathian, Edwin R van Teijlingen

**Affiliations:** 1 Academic Research Associate, Trauma Surgery Section, Surgery Department, Hamad General Hospital, Doha, Qatar; 2 Faculty of Health & Social Sciences, Bournemouth University, Bournemouth, UK.

Accurately predicting earthquakes is scientifically not possible. The seismology community argues that earthquakes are not predictable on a time scale useful for affected communities. Seimologists study the zones prone to earthquakes and estimate the most possible magnitude that is expected from active geological structures. But the current statistical models cannot predict the time and magnitude of any earthquake. Seismologists use arguments mainly grounded in non-linear physics and based on a different time scale, hence they can predict that it is highly likely that there will be a major earthquake in Nepal in the next 50-250 years [1].

In this sense the discipline of seismology is not that different from public health. In public health we can predict that heavy smokers are more likely to die early, especially from diseases such as lung cancer, but this does not mean that we can predict that (a) every smoker will die early; (b) get lung cancer; or (c) that non-smokers won’t get lung cancer.

Globally, the most frequently used four statistical models for the earthquake process are empirical studies on aftershock statistics, trigger models, epidemic type models, and parameterization of the models [1]. Yadav and colleagues have recently published about probabilistic estimates of the most perceptible earthquake magnitudes in the Himalayas and adjoining regions [2]. There is an urgent need of earthquake forecasting model for Nepal in this current scenario. It can be developed by the scientists of Nepal with the help of experienced international scientists. This will help the Nepalese to take timely and necessary precautions. We would argue that above all we need to use earthquake prediction knowledge to improve the disaster prepardness in local communities, service providers (hospitals, Non-Governmental Organizations, police, etc.), government policy-makers and international agencies [3]. On the whole, both seismology and public health are most successful when focusing on prevention not on prediction per se.
